# *Chlamydia trachomatis* serovars in urogenital and ocular samples collected 2014–2017 from Austrian patients

**DOI:** 10.1038/s41598-019-54886-5

**Published:** 2019-12-04

**Authors:** Iwona Lesiak-Markowicz, Anna-Margarita Schötta, Hannes Stockinger, Gerold Stanek, Mateusz Markowicz

**Affiliations:** Institute for Hygiene and Applied Immunology, Centre for Pathophysiology, Infectiology and Immunology, Medical University of Vienna, Vienna, Austria

**Keywords:** Clinical microbiology, Epidemiology

## Abstract

Infection of humans with *Chlamydia trachomatis*, a bacterial pathogen with a unique intracellular replication cycle, may cause a variety of clinical manifestations. These are linked to various serovars of the pathogen; trachoma to serovars A-C, oculogenital infections to serovars D-K, and lymphogranuloma venereum to serovars L1-L3. Nineteen serovars are known as human pathogens. The aim of the study was to determine the serovars of 401 *C. trachomatis* DNA positive extracts from original clinical specimens of patients in Austria including cervical and urethral swabs, urine, genital secretions and conjunctival swabs - collected from 2014 to 2017. Sequence analysis of the omp1 gene, encoding major outer-membrane protein was performed on each sample. In 50.1% of samples serovar E was identified and serovars F, D/Da and G/Ga were found in 16.2%, 9.7% and 9.0%, respectively. Remaining serovars were J (6.0%), K (4.7%), H (2.7%), B/Ba (1.0%), and I/Ia (0.5%). In 19 patients follow up samples could be tested. The majority of *C. trachomatis* serovars were associated with urogenital tract infections (D-K), however, one of them – serovar B/Ba - is linked to both, ocular and genital tract infection.

## Introduction

Sexually transmitted infections in the industrialised world are most commonly caused by *Chlamydia trachomatis*. According to WHO, about 130 million new cases occur each year worldwide^1^. Chlamydial urogenital infection mainly affects sexually active young people between the ages of 15 and 24 years^2^. Genital chlamydial infections are asymptomatic in a higher proportion in women than in men; thus, in women, chlamydiae can affect the upper genital tract causing inflammation and scarring, possibly followed by infertility, ectopic pregnancy and pre-term delivery^3^. Epididymitis, urethral obstructions and decreased fertility may occur in men. Infection with *C. trachomatis* also facilitates the transmission of HIV and is associated with cervical cancer^4^.

Currently, 19 serovars of *C. trachomatis* are recognised (A, B/Ba, C, D/Da, E, F, G/Ga, H, I/Ia, J, K, L1, L2, L2a and L3) according to specific epitopes of the major outer membrane protein (MOMP) encoded by the gene *omp1*. This gene encodes highly conserved protein structures that contain four evenly spaced domains whose sequences vary among the different serovars^5^. Classification of *C. trachomatis* isolates on the basis of the MOMP variable domains correlates with immunotyping using MOMP-specific monoclonal antibodies^6^. Serovars A, B, Ba and C are recognised as the aetiological agents of trachoma, serovars D–K are linked to oculogenital infections, and serovars L1–L3 are commonly associated with lymphogranuloma venereum.

Diagnosis of *C. trachomatis* infection is well established in Austria; however, little is reported on the occurrence and distribution of the serovars. We therefore analysed *C. trachomatis* serovars in DNA extracts from clinical samples submitted to our Institute by Austrian laboratories.

The study was approved by the Ethics Committee of the Medical University of Vienna, (EK-Number 1557/2017). Because of the retrospective design of the study, the Ethics Committee waived the need for informed consent.

## Results

**Figure 1:**
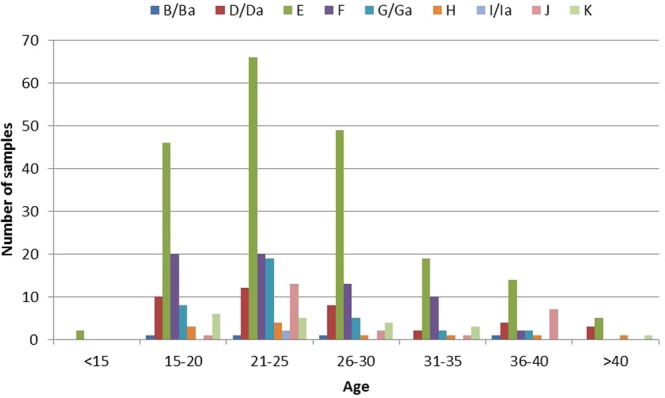
Age groups of patients and corresponding serovar distribution of 401 Chlamydia trachomatis samples.

Sequence analysis of the *omp1* gene fragments showed that among the 401 *C. trachomatis* samples, serovar E was the type most frequently identified (201/401, 50.1%), followed by serovars F (65/401, 16.2%), D/Da (39/401, 9.7%), G/Ga (36/401, 9.0%), J (24/401, 6.0%), K (19/401, 4.7%), H (11/401, 2.7%), B/Ba (4/401, 1.0%) and I/Ia (2/401, 0.5%). Information on the serovars and corresponding clinical samples are given in the Table 1. We identified nine different serovars in samples from female patients and five from male patients. The majority of samples were obtained from the urogenital tract and included samples of urine. Ten samples were derived from ocular swabs, including one from a new born infant. *C. trachomatis* was repeatedly detected in 19 patients, the time intervals between the first and the follow-up examinations ranging from 19 to 919 days. Sixteen patients had one follow-up visit, two patients had three and one patient had four. In 17 patients the same *C. trachomatis* serovar was identified in the first and follow-up sample: serovar E was detected the most frequently (8 patients), followed by serovar D/Da (3 patients) and J (2 patients); serovars B/Ba, F, G/Ga and I/Ia were found repeatedly in one patient each. In two patients different serovars were found in the follow-up samples; in one patient serovar E followed serovar K, and in the other serovar H followed serovar E. With regard to patient age, the frequency of *C. trachomatis* infection was highest in patients aged 21–25 years (143 samples), followed by those aged 15–20 (95 samples) and 26–30 (84 samples) (Fig. 1). Serovar E was the most common in all the age groups, followed by serovars F and G/Ga. There were only two positive samples from patients aged below 15 years: one ocular infection in a newborn (serovar E), one genital infection in a teenager (serovar B/Ba).

**Table 1:** Serovar distribution in 401 *Chlamydia trachomatis* samples from female and male patients.

	Specimens/Serovars	B/Ba	D/Da	E	F	G/Ga	H	I/Ia	J	K	Total
Female	Genital/Urethral	4	37	183	60	36	10	2	20	19	371
	Urine		1	1							2
	Ocular			6	1						7
Male	Genital/Urethral			7	2		1		3		13
	Urine		1	3	1						5
	Ocular			1	1				1		3

## Discussion

In 401 clinical specimens from Austrian patients who tested positive for *C. trachomatis*, *omp1* PCR identified serovar E as the most common type (50.1%), followed by serovars F, D/Da, G/Ga, J, K, H, B/Ba and I/Ia. The serovars D–F are recognised as the most prevalent worldwide^2,3,7,8^. Serovar B/Ba, detected in a genital sample, was the only serovar from the ocular group of *C. trachomatis*; this serovar has already been described as causing ocular and genital tract disease^9^. The majority of samples tested were derived from female patients (95%), where the most frequent serovars were E and F; these were also prominent in the small number of samples from male patients. Serovar E was also detected in an ocular swab of a newborn.

We did not find serovars L1-L3 in our mainly female study population.

Our aim was to get a first impression about *C. trachomatis* serovars obtained from clinical samples of Austrian patients. We identified eight different serovars of the oculogenital group, together with serovar B/Ba, one of the trachoma group and known as an agent of genital tract infection. We considered sequencing of the *omp1* gene fragment by using Sanger sequencing an efficient method for this purpose. Multilocus sequence typing and next generation sequencing of *C. trachomatis* would be the next step to analyse the strains in more detail in order to compare their relation to other isolates and in respect to clinical manifestations.

Our findings might serve as an incentive for future surveillance of *C. trachomatis* infection in Austria. Further, knowledge of the *C. trachomatis* serovar is relevant for deciding about the length of treatment of a *C. trachomatis* infection; e.g. 21 days for *C. trachomatis* serovars L1-L3^10^. Finally, it might have an impact on the selection of suitable immunogenic antigens for vaccine development.

## Materials and Methods

A total of 401 clinical specimens were analysed, originating from 378 patients (359 females and 19 males). In 19 patients (18 females and 1 male) repeated samples were taken - between 2 and 4 times per patient. Most of the specimens were genital/urethral samples. Under inclusion of retested patients, 371 samples were from female patients with a median age of 26 years (range 15–65 years), and 13 samples from male patients with a median age 36 years (range 23–42 years). Seven specimens were urine samples from 2 female patients (median age 24 years, range 22–26 years), 5 from male patients (median age 27 years, range 22–37 years), and 10 were ocular swabs derived from 7 female patients (median age 20 years, range 0.1–32 years) and 3 male patients (median age 32 years, range 28–39 years).

The PEQGoldTissue DNA Mini Kit (Peqlab, VWR, Darmstadt, Germany) was used for extraction of *C. trachomatis* DNA from the clinical samples. The DNA was stored at −20 °C until serovar determination.

The *omp1* gene fragment (ca. 1130 bp) was amplified in nested PCR according to a method described by Hsu *et al*.^11^. Briefly, the outer primer pair NLO/NRO was used for primary amplification and the inner primer pair MOMP87/C214 for the second run, to obtain the 584 bp products. The final PCR products were analysed on 1% agarose gel using GelRed® Nucleic Acid Gel Stain (Biotium, Germany). It should be noted that serovar subtypes Da, Ba, Ga and Ia cannot be discriminated using this method.

The *omp1* fragments were purified from the gel using the QIAquick Gel Extraction Kit (Qiagen, Hilden, Germany). DNA concentration was then measured (DeNovix Spectrophotometer/Fluorometer), followed by bi-directional sequencing of the samples (Microsynth, Vienna, Austria) using the primer set MOMP87/C214. Consensus sequences were compared with sequences of known *C. trachomatis* strains by BLAST search provided by NCBI (www.ncbi.nlm.nih.gov/GenBank).

## Data Availability

All data generated or analysed during this study are included in this published article.
